# Skeletal muscle metabolic gene response to carbohydrate feeding during exercise in the heat

**DOI:** 10.1186/1550-2783-10-40

**Published:** 2013-09-13

**Authors:** Charles L Dumke, Dustin R Slivka, John S Cuddy, Walter S Hailes, Brent C Ruby

**Affiliations:** 1 Department of Health and Human Performance, Montana Center for Work Physiology and Exercise Metabolism, University of Montana, 32 Campus Drive, Missoula, MT 59812, USA; 2 School of Health, Physical Education and Recreation, University of Nebraska at Omaha, 6001 Dodge Street, Omaha, NE 68182, USA

**Keywords:** Mitochondrial biogenesis, PGC-1α, UCP3, Carbohydrate supplementation, Substrate utilization

## Abstract

**Background:**

Heat stress down-regulates mitochondrial function, while carbohydrate supplementation attenuates the exercise induced stimulation of mitochondrial biogenesis in humans. The effects of exogenous carbohydrate during exercise in the heat on metabolic mRNA have not been investigated in humans. The purpose of this study was to determine the impact of exercise with and without carbohydrate supplementation on skeletal muscle metabolic response in the heat.

**Methods:**

Eight recreationally active males (4.05 ± 0.2 L^.^min^-1^) completed 2 trials which included 1 hr of cycling at 70% workload max and 3 hr recovery in a hot environment. Both trials were conducted in a climate controlled environmental chamber (38°C and 40% RH). The trials differed by the consumption of either a 6% carbohydrate (CHO) containing beverage (8 ml^.^kg^-1.^hr^-1^) or placebo (P) during exercise in random order. Muscle biopsies were obtained from the *vastus lateralis* before exercise, immediately post-exercise and at the end of the 3 hr recovery period. Muscle was analyzed for muscle glycogen and mRNA related to metabolic and mitochondrial development (MFN2, PGC-1α, GLUT4, UCP3). Expired gases were measured to determine whole body substrate use during exercise.

**Results:**

Carbohydrate oxidation and muscle glycogen utilization did not differ between trials, whereas fat oxidation was elevated during exercise in P. Exercise caused an increase in PGC-1α, and GLUT4 (P < 0.05) independent of exogenous carbohydrate provision. Carbohydrate consumption attenuated the mRNA response in UCP3 (P < 0.05).

**Conclusions:**

This study indicates that the provision of exogenous carbohydrate attenuates the stimulation of mRNA expression of UCP3 following exercise in the heat.

## Background

Mitochondrial adaptation is recognized as important in both health and disease. For some time it has been known that exercise induces critical adaptations in mitochondrial function within skeletal muscle [1]. More recently other factors have been considered key modifiers of mitochondrial and metabolic adaptation such as fat feeding [2], select bioflavonoids [3,4], intensity, duration and frequency of exercise [5-8], environmental temperature [9-13], and carbohydrate availability during exercise [14-16]. Acute markers for mitochondrial and metabolic alterations in fuel oxidation used in these investigations include mRNA for many different proteins involved in metabolism. These genes include the transcription regulator mitochondrial development peroxisome-proliferator- activated receptor-gamma co-activator 1 alpha (PGC-1α) [17,18], uncoupling protein 3 (UCP3) [12,19,20], the mitochondrial fusion protein mitofusin 2 [12,21], and other metabolic genes important for carbohydrate oxidation such as glucose transporter 4 (GLUT4) [22].

Carbohydrate consumption during exercise is capable of altering the stimuli for metabolic adaptation [14-16]. Cluberton et al. [14] have shown that carbohydrate consumption during exercise can attenuate the metabolic gene expression when completed in ambient temperatures. They showed that consumption of a 6% carbohydrate beverage during 1 hr of cycling at ~74% VO_2max_ lowered the exercise induced increase in mRNA of PDK4 and UCP3 3 hr post-exercise, but not PGC-1α or GLUT4. As the authors suggest, this attenuation may be due to the increase in carbohydrate oxidation, suppression of circulating free fatty acids, and the elevation of insulin by exogenous carbohydrate consumption. Similar to carbohydrate consumption during exercise, exposure to heat in exercising humans has been shown to result in an upregulation of carbohydrate oxidation [23,24]. How carbohydrate delivery in the heat affects the metabolic adaptation to exercise remains poorly understood.

Previously we have shown in humans that PGC-1α gene expression is elevated in the cold, and attenuated following exercise in hot environments [12]. We demonstrated a ~20% reduction in PGC-1α mRNA following exercise in the heat (33°C). This attenuation in the heat is supported in other models as heat stress down-regulates mitochondrial function in yeast and broiler chickens [9-11]. In yeast, microarray genes associated with mitochondrial respiration were depressed following exposure to mild heat stress (37°C), and conversely genes associated with glycolysis were upregulated [10]. However this is not a universal finding across different experimental models [13,25]. In the absence of exercise, heat is capable of elevating expression of UCP3 in porcine muscle [25].

Since both environmental temperature and substrate availability can alter the metabolic gene response to exercise [12,14], it remains to be seen if carbohydrate ingestion in the heat attenuates the metabolic gene response following exercise and recovery in humans. Our purpose was to determine the impact of carbohydrate supplementation on select markers of exercise induced metabolic mRNA (PGC-1α, MFN2, UCP3, and GLUT4) in a hot environment (38°C).

## Methods

### Subjects

Eight male participants (23.5 ± 1.4 yrs, 76.6 ± 1.7 kg, 52.9 ± 2.2 ml•kg^-1^•min^-1^, 12.4 ± 1.6% body fat) volunteered for participation in the study. Prior to testing, participants read and signed an informed consent form approved by the University of Montana Institutional Review Board for the ethical use of human subject research and meet the standards of the Declaration of Helsinki.

#### *Experimental design*

Subjects (N = 8) completed 2 trials of 1 hr cycling at a constant load of 70% workload max (195.6 ± 11.3 watts) and 3 hr of recovery in a hot environment. Subjects arrived in the morning following an 8 hr fast. Both trials were in a controlled climate chamber at 38°C and 40% relative humidity, and separated by at least one week. Subjects were not heat acclimatized since the study was conducted in April at ~46°N latitude at the end of the northern hemisphere winter. The two counterbalanced trials for each participant differed by the provision of either a 6% carbohydrate (CHO) or placebo (P) beverage in random order. To achieve a 6% CHO solution, maltodextrin was mixed with an artificially flavored and sweetened commercially available powder (Crystal Light, Kraft Foods, Glenview, IL). Placebo contained the commercially available powder with no maltodextrin or other macronutrient energy, both P and CHO included 140 mg sodium per liter. Subjects were instructed to abstain from strenuous exercise for 48 hr, and no exercise for 24 hr before each trial. Subjects recorded diet intake for 24 hr prior to the day of the first trial and were instructed to replicate this exact diet prior to the second trial day. Muscle biopsies were collected pre ride, post ride and at the end of the 3 hr of recovery. On the morning of the trials, immediately prior to the exercise bout (< 5 min) subjects ingested 8 ml•kg^-1^ of the prescribed beverage, during exercise each beverage was consumed at a rate of 4 ml•kg^-1^•30 min^-1^ (~37 g•hr^-1^ for CHO trial) and 4 ml•kg^-1^•hr^-1^ (~18.4 g•hr^-1^ for CHO trial) during recovery. Body weights were recorded prior to entering the climate chamber, post ride, and at the end of the 3 hr recovery. Core temperatures were not measured since the chamber temperature was the same for both trials. Previously published reports from our lab indicate that a similar exercise protocol in the heat results in rectal temperatures exceeding 39°C [26]. Expired gases and rating of perceived exertion (RPE) were measured at 4, 24, and 54 min during the 1 hr exercise. VO_2_ and VCO_2_ were used determine whole-body fuel oxidation using the equation of Péronnet and Massicotte [27].

#### *Body composition*

Body density was determined using hydrodensitometry and corrected for estimated residual lung volume. Net underwater weights were recorded using load cells (Exertech, Dresbach, MN). Body density was then converted to body composition using the Siri equation [28].

#### *Maximal exercise capacity*

Maximum oxygen consumption (VO_2_max) and power associated with VO_2_max was measured for each fasted subject using a graded exercise protocol (starting at 95 W and increasing 35 W every three minutes) on an electronically braked cycle ergometer trainer (Velotron, RacerMate Inc., Seattle, WA). Maximum power was calculated as the highest completed stage (in W) plus the proportion of time in the last stage multiplied by the 35 W stage increment. Expired gases were measured and averaged in 15-second intervals during the test using a calibrated metabolic cart (Parvomedics, Inc., Salt Lake City, UT).

#### *Biopsies*

Biopsies were obtained pre and post exercise and following 3 h of recovery for the analysis of muscle glycogen, and metabolic gene expression (see below). Biopsies were taken from the *vastus lateralis* muscle using a 4–5 mm Bergstrom percutaneous muscle biopsy needle with the aid of suction. Biopsies were obtained from the same leg for a given trial using a separate incision 2 cm proximal to the previous biopsy. After excess blood, connective tissue, and fat were quickly removed, tissue samples (50–100 mg) were immersed in liquid nitrogen and stored at −80°C for subsequent analysis.

#### *Glycogen*

Muscle glycogen was analyzed using an enzymatic spectrophotometric method. Muscle samples were weighed (5–15 mg) upon removal from a −80°C freezer and placed in 0.5 ml, 2 N HCl solution. The sample solutions were weighed, incubated for two hours at 100°C in an oven, then re-weighed and re-constituted to their original weight using distilled water. To normalize pH, 1.5 ml of 0.67 M NaOH was added. An aliquot of this muscle extract (100 μl) was added to 1 ml of Infinity glucose (HK) liquid stable reagent (Thermo Fisher Scientific, Waltham, MA) and the absorbance read on a spectrophotometer at 340 nm. Glycogen concentration was calculated using the extinction co-efficient of NADH. Muscle glycogen concentrations are expressed in mmol ⋅ kg^-1^ wet weight of muscle tissue.

#### *mRNA isolation*

An 8–20 mg piece of skeletal muscle from the pre-exercise and 3 h recovery biopsies was homogenized in 800 μl of trizol (Invitrogen, Carlsbad CA, Cat# 15596–018) using an electric homogenizer (Tissue Tearor, Biosped Products Inc, Bartlesville OK). Samples were then incubated at room temperature for 5 minutes after which 200 μl of chloroform per 1000 μl of trizol was added and shaken vigorously. After an additional incubation at room temperature for 2–3 minutes the samples were centrifuged at 12,000 g for 15 minutes and the aqueous phase was transferred to a fresh tube. mRNA was precipitated by adding 400 μl of isopropyl alcohol and incubated overnight at −20°C. The next morning samples were centrifuged at 12,000 g for 10 minutes at 4°C and the mRNA was washed by removing the supernatant and adding 800 μl of 75% ethanol. Samples were vortexed and centrifuged at 7,500 g for 5 minutes at 4°C. mRNA was re-dissolved in 100 μl RNase-free water after the supernatant was removed and the mRNA pellet was dried. The RNA was cleaned using the RNeasy mini kit (Qiagen, Valencia CA, Cat#74104) according to the manufacturer’s protocol using the additional DNase digestion step (RNase-free DNase set, Qiagen, Valencia CA, Cat# 79254). RNA purity was analyzed by the A260:A280 ratio and quantified on a nano-spectrophotometer (nano-drop ND-1000, Wilmington DE).

*cDNA synthesis*. First-strand cDNA synthesis was achieved using Superscript-first-strand synthesis system for RT-PCR kit (Invitrogen, Carlsbad CA, Cat #11904-0818) according to the manufacturer’s protocol. Each sample within a given subject was normalized to the same amount of RNA. The resulting cDNA was then diluted two fold using RNase free water in order to have adequate volume for RT-PCR and frozen for later analysis.

#### *Real time RT-PCR*

Primer and Probe sequences are presented in Table 1. Each 25 μl reaction volume contained 500 nM primers, 250 nM probe (PrimeTime qPCR assay, Integrated DNA technologies), 1× FastStart TaqMan Probe master (Roche Applied Science, Indianapolis IN), and 2.5 μl of sample cDNA. PCR was then run using the Bio-Rad I Cycler iQ5 Real-Time PCR Detection system (Bio-Rad, Hercules CA) using a 2-step Roche protocol (1 cycle at 50°C for 10 minutes, 1 cycle at 95°C for 10 minutes, followed by 40 cycles of 95°C for 15 seconds followed by 60°C for 1 minute). Quantification of mRNA from the pre and 3 h post exercise samples was calculated using the 2^-ΔΔCT^ as described earlier [29,30]. GAPDH was used as the reference housekeeping gene as it has been demonstrated to be the most stable among other common housekeeping genes following aerobic exercise and environmental temperature [12,31,32]. The stability of GAPDH was analyzed by the ΔCT method [29,30].

**Table 1:** Primers and probes used for real-time PCR

**Gene**	**Primer 1**	**Primer 2**	**Probe**
GAPDH	TGTAGTTGAGGTCAATGAAGGG	ACATCGCTCAGACACCATG	AAGGTCGGAGTCAACGGATTTGGTC
MFN2	ATGCATCCCACTTAAGCAC	CCAGAGGGCAGAACTTTCTC	AGAGGCATCAGTGAGGTGCT
PGC-1α	ATAAATCACACGGCGCTCTT	TGAGAGGGCCAAGCAAAG	AGAGGCAGAGGCAGAAGG
UCP3	CAAAATCCGGGTAGTGAGGCT	TGACTCCGTCAAGCAGGTGTAC	CCCCCAAAGGCGCGGACAAC
GLUT4	TCTTCACCTTGGTCTCGGTGTTGT	CACGAAGCCAAAGATGGCCACAAT	ATGTGTGGCTGTGCCATCCTGATGA

*GAPDH* Glyceraldehyde 3-phosphate dehydrogenase, *MFN2* mitofusin 2, *PGC*-*1α* peroxisome-proliferator- activated receptor-gamma co-activator 1 alpha, *UCP3* uncoupling protein 3, *GLUT4* glucose transporter 4.

#### Statistics

Dependent variables were compared using two-way repeated-measures ANOVA’s (time x trial or exercise-recovery × CHO). In the event of a significant f-ratio, post hoc Fishers protected least significant difference procedure was used to determine where differences occurred. All statistics were performed using SPSS for windows Version 13 (Chicago, IL). A probability of type I error less than 5% was considered significant (p < 0.05). All data are reported as mean ± SE.

## Results

### Exercise trials

Prescribed fluid intakes were 2.16 ± 0.05 L over the course of the one hour of exercise and 3 h of recovery. Subjects lost an average of 0.63 ± 0.07 and 0.73 ± 0.13 kg body weight during the CHO and P trials respectively (p < 0.05), regardless of trial. This <1% of body weight loss suggests fluid intakes were sufficient to adequately meet sweat rates during the hot trials. The prescribed carbohydrate intake amounted to 129.6 ± 3.0 g of carbohydrate, or 518.4 ± 12.0 kcals over the 4 hr in the climate chamber during the CHO trial. Heart rate, RPE, oxygen consumption and carbon dioxide production increased during the exercise period (p < 0.05), but did not differ between trials (Table 2).

**Table 2:** **VO**_**2**_**, VCO**_**2**_**, RER, HR, and RPE during 1 h exercise trials in the heat, with and without CHO**

	**Placebo**			**CHO**		
	**4 min**	**24 min**	**54 min**	**4 min**	**24 min**	**54 min**
**VO**_**2 **_**(L · min**^**-1**^**)**	2.91 ± 0.10	3.21 ± 0.15	3.63 ± 0.19^*^	3.01 ± 0.16	3.25 ± 0.16	3.52 ± 0.22^*^
**VCO**_**2 **_**(L · min**^**-1**^**)**	2.64 ± 0.07	2.79 ± 0.12	3.11 ± 0.17^*^	2.72 ± 0.13	2.87 ± 0.15	3.10 ± 0.19^*^
**RER**	0.91 ± 0.01	0.87 ± 0.01	0.86 ± 0.01^†^	0.90 ± 0.01	0.88 ± 0.01	0.88 ± 0.01^†^
**HR (beats · min**^**-1**^**)**	138.5 ± 6.7	158.9 ± 5.4	172.6 ± 4.9^*^	151.4 ± 5.9	162.0 ± 5.4	173.1 ± 4.4^*^
**RPE**	12.6 ± 0.3	15.0 ± 0.5	17.8 ± 0.6^*^	12.4 ± 0.5	15.1 ± 0.5	17.9 ± 0.4^*^

^*^p < 0.05 main effect of time; ^†^ p < 0.05 main effect of trial X time.

**Figure 1:**
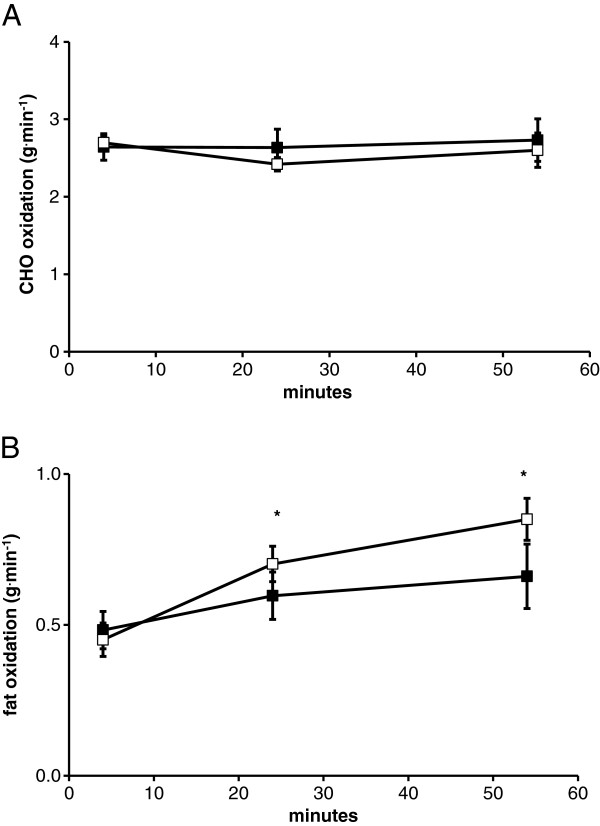
**Substrate oxidation during exercise in the heat. A**. represents carbohydrate oxidation for 1 hr in the heat with gas measurements made at 4, 24, and 54 min. **B**. represents fat oxidation for 1 hr in the heat with gas measurements made at 4, 24, and 54 min. Open and solid symbols represent the P and CHO trials respectively. * - indicates a significant main effect of time.

Subjects finished the exercise trial at a mean RPE of >17 (Table 2), suggesting that the combination of the heat and exercise was perceptually difficult. RER was lower by the end of the 1 hr exercise bout during P compared to CHO trial (significant trial × time interaction, p = 0.017), demonstrating a greater reliance on fat by the end of the P trial (Table 2). There was not a significant effect of exercise (p = 0.5) or trial (p = 0.18) on absolute carbohydrate oxidation (Figure 1A). Absolute fat oxidation was not different between trials (p = 0.10), but did show a significant increase (p = 0.02) in fat use by the end of their 1 hr bout of cycling (Figure 1B).

**Figure 2:**
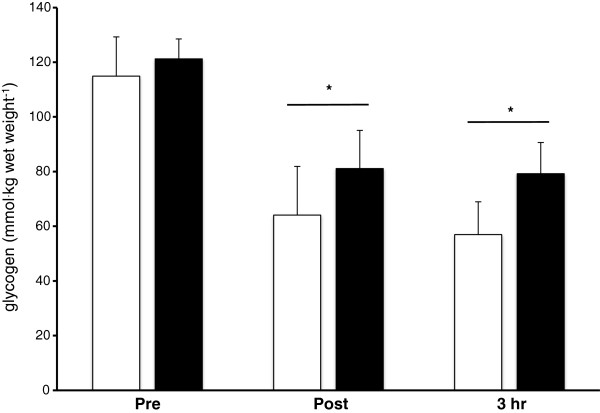
**Muscle glycogen concentration pre, post-exercise and following 3 hr of recovery.** Open and solid bars represent the P and CHO trials respectively. * - indicates a significant main effect of time.

*Muscle Glycogen* Muscle glycogen did not differ between trials (p = 0.57), but decreased as a result of the exercise bout (p < 0.001) (Figure 2). This represents a 35% and 44% reduction pre and post exercise for the CHO and P trial respectively. Muscle glycogen did not significantly increase from post exercise to 3 hr of recovery in either trial.

**Figure 3:**
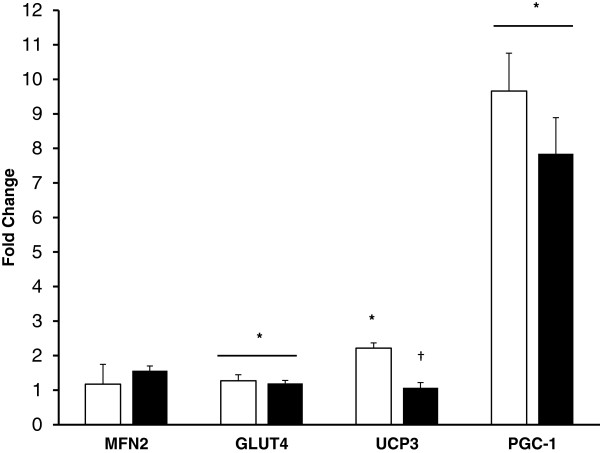
**Muscle expression for metabolic and mitochondrial genes following 3 hr of recovery post-exercise.** Open and solid bars represent the P and CHO trials respectively. * - indicates a significant main effect of time, and ^†^ - indicates a significant trial X time interaction.

*Gene Expression* There was not a significant effect of exercise in the heat on our housekeeping gene, GAPDH (p = 0.3). Metabolic and mitochondrial gene expression from the pre and 3 hr post exercise muscle samples using the 2^-ΔΔCT^ method is presented in Figure 3. There was a significant effect for exercise on GLUT4 mRNA (P = 0.04), increasing 20% and 27% in the CHO and P trial respectively. GLUT4 expression was not altered by CHO treatment. Exercise increased PGC-1α (P < 0.001) 8 and 9.5 fold in the CHO and P trial respectively, but did not show a significant effect of treatment (P = 0.15). MFN2 did not change with exercise in the heat or carbohydrate supplementation. There was a significant effect of exercise (P < 0.001) and interaction (CHO × exercise) for UCP3 (P = 0.001), where UCP3 mRNA in the placebo trial increased over 2 fold but did not increase during the CHO trial.

## Discussion

These data support previous research demonstrating the carbohydrate attenuation of metabolic adaptations to exercise. Specifically, this investigation showed the attenuation of the exercise stimulation of skeletal muscle UCP3 mRNA with carbohydrate consumption in the heat. We also confirmed exercise induced increases in GLUT4 and PGC-1α in the heat. A previous investigation demonstrated that carbohydrate consumption during exercise attenuated the mRNA expression for both UCP3 and PDK4, and only a trend towards GLUT4 in ambient conditions [14]. Similarly, we did not show a significant effect of carbohydrate consumption on GLUT4 (p = 0.7), but did observe an attenuation in UCP3 mRNA in the current investigation. A direct comparison between environmental temperatures would need to be performed to determine if environmental conditions alter these CHO attenuating effects.

In the current investigation carbohydrate oxidation did not differ between trials despite exercising for 1 hr at 70% workload max at 38°C and 40% RH with and without oral carbohydrate consumption. Perhaps the similar rates of carbohydrate oxidation are due to an increase in the oxidation of endogenous carbohydrate in the heat during the P trial. Our selection of study design does not allow us to make this direct comparison, however the increase in carbohydrate oxidation in the heat is well established [23,24]. This may explain why only UCP3 was attenuated in the CHO trial in our investigation and not GLUT4. The glucose transporter GLUT4 is a gene linked to carbohydrate oxidation [33,34]. Cluberton et al. [14] showed a trend (p = 0.09) for carbohydrate consumption to attenuate the exercise induced increase in gene expression for GLUT4 under ambient conditions. Although they demonstrated a 2 fold increase with exercise on GLUT4 expression, it is not apparent that this reached statistical significance. In the current study, although there was a significant effect of exercise, we saw no evidence of carbohydrate ingestion on GLUT4 mRNA expression (p = 0.7). It is compelling to believe that this may be due to the lack of difference between CHO and P trials in absolute carbohydrate oxidation in the heat, which may mask the effects of carbohydrate ingestion on this gene. It is a limitation of the current study that there were not ambient temperature trials (with and without carbohydrate) by which to compare the effects of the heat, however this was eliminated due to the stress on the subjects (amounting to 4 trials and 12 biopsies). The purpose of this study was to provide initial insight on this topic, while relying on the previously reported effect of CHO consumption under ambient conditions on these genes [14].

Depletion of glycogen is thought to be a potential aspect of the stimulation of mitochondrial biogenesis [35]. Exercise in the current study was sufficient to lower muscle glycogen levels ~40%, which is believed to be capable of stimulating AMPK, an upstream covalent modifier of PGC-1α [5,36,37]. In the current study glycogen depletion and carbohydrate oxidation did not differ between trials during the 1 h of exercise, indirectly suggesting that AMPK activity was similar between trials. This is supported by others, as carbohydrate ingestion during cycling is not thought to alter glycogen utilization [14,38]. As well, carbohydrate ingestion during cycling does not appear to alter AMPK signaling in humans [39]. This may explain why GLUT4 was not different between trials, since AMPK is thought to be a potent simulator of GLUT4 transcription [40]. Despite this lack of effect of carbohydrate ingestion on GLUT4, UCP3 mRNA expression was attenuated by carbohydrate ingestion. This suggests that the UCP3 gene may be more sensitive to fat oxidation. We showed a significant effect of carbohydrate ingestion on RER, with the P trial demonstrating greater fat reliance by the end of the exercise bout. We unfortunately do not have substrate oxidation data for the 3 h of recovery prior to the last biopsy, when mRNA expression was sampled. However since the P trial received no carbohydrate into the recovery period, it is quite possible that the greater fat oxidation during the later stages of exercise continued into recovery in the P trial and subsequently attenuated the UCP3 mRNA expression. This is supported by evidence that elevated circulating fatty acids are associated with the upregulation of skeletal muscle expression of UCP3 [14,41-43]. We do not have evidence of circulating free fatty acids (FFA) in the current study, but it is well established that fasted exercise in the absence of carbohydrate delivery elevates FFA compared to carbohydrate trials [44]. Although fat oxidation appears to coincide with UCP3 expression, the metabolic role of this protein in skeletal muscle remains unclear as it suggests a loss of exercise efficiency by uncoupling the proton gradient created in the electron transport chain from ATP synthesis. However, besides fat oxidation, UCP3 has been implicated as being important in the control of thermogenesis and the regulation of oxidative stress [45]. The long term implications of the attenuation of UCP3 expression following exercise with carbohydrate supplementation in this study and others has yet to be determined [14,43]. It is intriguing to think that lower UCP3 mRNA may play a role in previous evidence of the carbohydrate attenuating effect on fat oxidation with exercise training [44,46]. These studies demonstrated that low carbohydrate availability (fat adapted) resulted in greater rates of fat oxidation even when glycogen levels were restored with a day on a high carbohydrate diet. Our study and others have shown that UCP3 is the gene most consistently attenuated with the consumption of exogenous carbohydrate. How UCP3 expression is affected during longer periods of low carbohydrate availability remain to be seen. Acute changes in mRNA expression must be interpreted with caution, since protein amounts as the result of chronic adaptation were not the focus of this study.

For the other genes investigated, this study is consistent with previous literature which shows that the expression of GLUT4 [22] and PGC-1α mRNA is elevated following exercise [6,17,18]. More surprisingly, exercise stimulated increases in mRNA were not seen in MFN2, as these have previously been shown to be sensitive to exercise [8,12,14,21,47]. We confirmed in this study that our housekeeping gene was insensitive to both heat and exercise, and this is supported in the literature [12,31,32]. Therefore, it remains unknown why an exercise induced increase in MFN2 was not observed in the current study. MFN2 is a mitochondrial membrane protein involved in the fusion events of the mitochondrial architecture [21]. Increased expression of this gene is thought to lead to greater mitochondrial function through matrix protein mixing [48]. One of our previous investigations showed robust (~50%) increases in MFN2 following 5 hr of cycling, suggesting that greater exercise intensity or duration may be needed for up regulation of this gene [8]. However, in another investigation from our lab, 1 hr of cycling at 60% of maximum workload increased MFN2 expression (~20%) [12]. In the current study the exercise protocol (1 hr at 70% maximum workload) should have been sufficient to increase MFN2 gene expression. Due to the design of this study it is not apparent whether this is due to the modest stress of the exercise bout, modest changes in individual variability in a somewhat small sample size, or an attenuating effect of the hot environment. We previously showed that MFN2 is not significantly affected by exercise in varying environmental temperatures, with similar exercise responses in the heat (33°C), cold (7°C), and neutral (20°C) environments [12]. This suggests that small increases in variability with a sample size of eight may have affected the statistical outcome of this particular gene. Despite this, carbohydrate supplementation had no apparent attenuating effects on this mitochondrial fusion gene. To our knowledge this is the first time MFN2 has been investigated following carbohydrate supplementation in humans.

## Conclusions

These data contribute to the general understanding of stimuli regulating metabolic adaptation following exercise. We found that exercise and recovery in the heat stimulates genes for PGC-1α, UCP3 and GLUT4. Carbohydrate ingestion during exercise and recovery in a hot environment attenuated mRNA expression of UCP3, but had no effect on the expression of MFN2, GLUT4 and PGC-1α. It remains to be seen through a direct comparison of environmental temperatures if this is due to similar carbohydrate oxidation rates when carbohydrate is ingested in the heat during exercise.

Regardless of environmental temperature, these data should not be interpreted as reason to avoid ingesting carbohydrate during exercise. Carbohydrate delivery during exercise bouts of >1 hr is well known to increase performance [49-51]. However, a growing body of evidence may also suggest that carbohydrate availability during training bouts can alter the metabolic response and perhaps result in increased reliance on fat stores when carbohydrate availability is low [2,7,8,52]. The concept of a ‘periodized diet’ to control and maximize fuel oxidation and the adaptations to specific blocks of training for both endurance and resistance exercise is an exciting new area of applied sport nutrition research.

## Abbreviations

CHO: Carbohydrate; P: Placebo; VO2: Oxygen consumption; VO2max: Maximal oxygen consumption; VCO2: Carbon dioxide production; RER: Respiratory exchange ratio; RPE: Rating of perceived exertion; mRNA: Messenger ribonucleic acid; PGC-1α: Peroxisome-proliferator- activated; receptor-gamma: Co-activator 1 alpha; UCP3: Uncoupling protein 3; MFN2: Mitofusin 2; GLUT4: Glucose transporter 4; GAPDH: Glyceraldehyde 3-phosphate dehydrogenase; PDK4: Pyruvate dehydrogenase 4.

## Competing interests

The authors declare that they have no competing interests in access to these data or associations with companies involved with products used in this research. This study was supported by a grant from the Office of Naval Research Grant Award N000140910850.

The views and conclusions contained herein are those of the authors and should not be interpreted as necessarily representing the official policies or endorsements, either expressed or implied, of the Office of Naval Research or the U.S. government.

## Authors’ contributions

CLD participated in conception, design, and data acquisition, assisted in PCR analysis and interpretation of data, and wrote the manuscript. DRS participated in conception, design, and data acquisition, assisted in PCR analysis and interpretation of data, and aided in the drafting and revising of the manuscript. JSC participated in data acquisition, analysis and interpretation of data, and aided in the drafting and revising of the manuscript. WSH participated in data acquisition, analysis and interpretation of data, and aided in the drafting and revising of the manuscript. BCR participated in conception, design, and data acquisition, assisted in analysis and interpretation of data, and aided in the drafting and revising of the manuscript. All authors have read and given final approval of this version of the manuscript for publication.
